# Supplementary material for: Venomix: a simple bioinformatic pipeline for identifying and characterizing toxin gene candidates from transcriptomic data
Source: PeerJ. 2018 Jul 31;6:e5361. doi: 10.7717/peerj.5361 (PMC6074769; doi:10.7717/peerj.5361)
Supplement: Supplemental Information 3 [file peerj-06-5361-s003.gz › FinalOutput_E-20/Plancitoxin-1_1/finaltree.pdf]

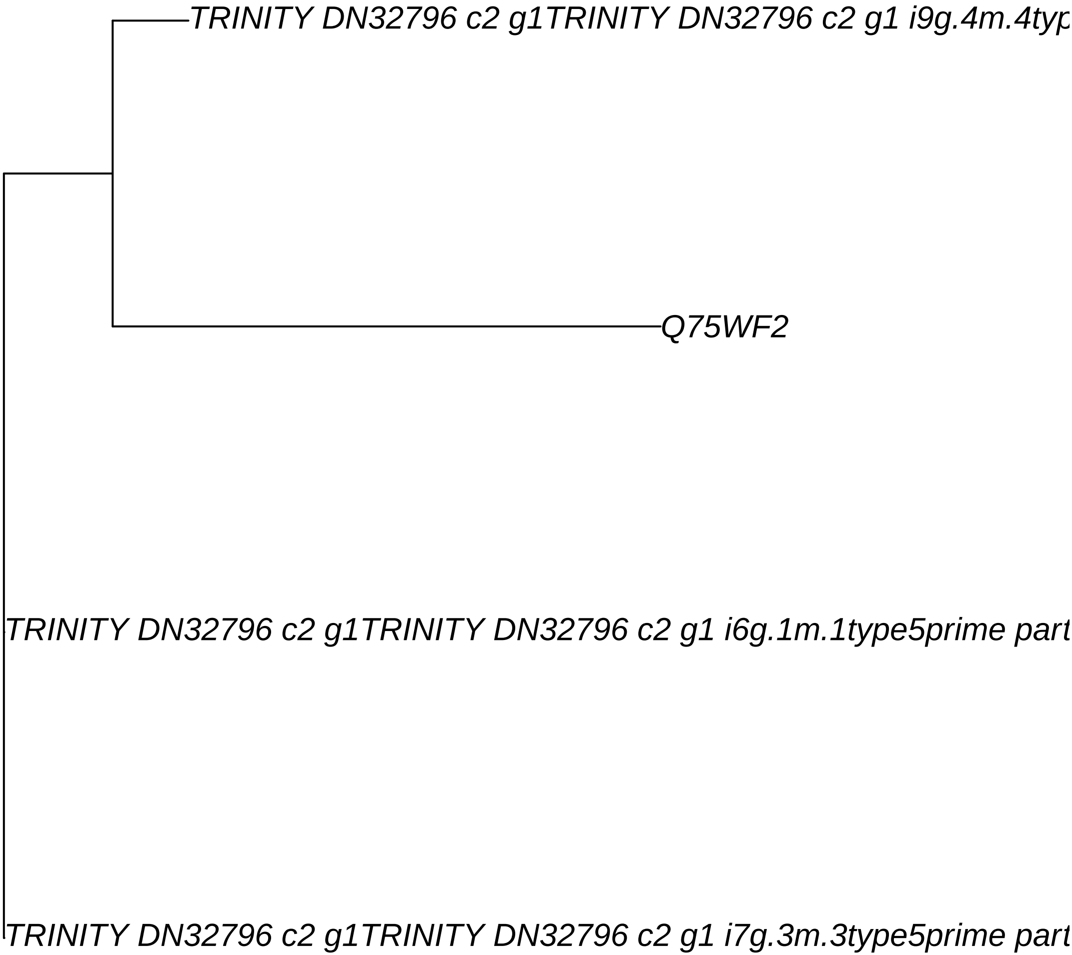

TRINITY DN32796 c2 g1TRINITY DN32796 c2 g1 i9g.4m.4typ
Q75WF2
TRINITY DN32796 c2 g1TRINITY DN32796 c2 g1 i6g.1m.1type5prime part
TRINITY DN32796 c2 g1TRINITY DN32796 c2 g1 i7g.3m.3type5prime part
